# Clinical significance of obstructive sleep apnea in patients with acute coronary syndrome with or without prior stroke: a prospective cohort study

**DOI:** 10.1186/s40001-023-01071-0

**Published:** 2023-03-01

**Authors:** Bin Wang, Wen Hao, Jingyao Fan, Yan Yan, Wei Gong, Wen Zheng, Bin Que, Hui Ai, Xiao Wang, Shaoping Nie

**Affiliations:** 1 Center for Coronary Artery Disease, Division of Cardiology, Beijing Anzhen Hospital, Capital Medical University, No. 2 Anzhen Road, Chaoyang District, Beijing, 100029 China; 2 National Clinical Research Center for Cardiovascular Diseases, No. 2 Anzhen Road, Chaoyang District, Beijing, 100029 China

**Keywords:** Acute coronary syndrome, Obstructive sleep apnea, Stroke, Outcome

## Abstract

**Background and objective:**

Whether obstructive sleep apnea (OSA) is associated with worse prognosis in patients with acute coronary syndrome (ACS) with or without prior stroke remains unclear. We investigated the association of OSA with cardiovascular events in ACS patients with or without prior stroke.

**Methods:**

Between June 2015 and January 2020, we prospectively recruited eligible ACS patients who underwent cardiorespiratory polygraphy during hospitalization. We defined OSA as an apnea hypopnea index (AHI) ≥ 15 events/hour. The primary composite end point was major adverse cardiovascular and cerebrovascular events (MACCEs), including cardiovascular death, myocardial infarction, stroke, ischemia-driven revascularization, or hospitalization for unstable angina or heart failure.

**Results:**

Among 1927 patients enrolled, 207 patients had prior stroke (10.7%) and 1014 had OSA (52.6%). After a mean follow-up of 2.9 years, patients with stroke had significantly higher risk of MACCEs than those without stroke (hazard ratio [HR]:1.49; 95% confidence interval [CI]: 1.12–1.98, *P* = 0.007). The multivariate analysis showed that patients with OSA had 2.0 times the risk of MACCEs in prior stroke group (41 events [33.9%] vs 18 events [20.9%]; HR:2.04, 95% CI:1.13–3.69, *P* = 0.018), but not in non-prior stroke group (186 events [20.8%] vs 144 events [17.4]; HR:1.21, 95% CI 0.96–1.52, *P* = 0.10). No significant interaction was noted between prior stroke and OSA for MACCE (interaction P = 0.17).

**Conclusions:**

Among ACS patients, the presence of OSA was associated with an increased risk of cardiovascular events in patients with prior stroke. Further trials exploring the efficacy of OSA treatment in high-risk patients with ACS and prior stroke are warranted.

*Trial registration* Clinicaltrials.gov identifier NCT03362385.

**Supplementary Information:**

The online version contains supplementary material available at 10.1186/s40001-023-01071-0.

## Background

Obstructive sleep apnea (OSA) is a complex and heterogeneous common chronic disease characterized by repetitive episodes of upper airway collapse, affecting 40%–60% of patients with ACS [1, 2]. Current evidence has shown that OSA initiates and exacerbates coronary atherosclerosis and is closely related to poor outcomes in patients with ACS or cerebrovascular disease [1, 3–6]. However, several randomized controlled trials have indicated that treatment with continuous positive airway pressure (CPAP) is not associated with lower rates of recurrent cardiovascular events in patients with ACS [7–10]. It is well known that ACS and stroke share common pathogeneses, such as lipid disorders and arterial thrombosis. For that reason, ACS patients presenting with a history of stroke were not uncommon, constituted 8.25% of patients, and presented a therapeutic conundrum [11]. Furthermore, patients with prior stroke had particularly poor outcomes compared with those without prior stroke, including a higher risk of death, MI, and stroke [11–15]. Preliminary evidence also suggests that OSA is an independent risk factor for stroke and is associated with recurrent ischemic stroke and worsening outcomes [16–18]. Although whether CPAP treatment reduces the risk of stroke in OSA patients remains controversial, patients’ adherent to CPAP therapy (> 4 h per day) may benefit [19]. In addition, it is unclear whether the effect of OSA on the prognosis of patients with ACS varies based on previous stroke. Considering the association between ACS, OSA and stroke, we hypothesized that OSA combined with prior stroke may have a synergistic deleterious effect that increases future cardiovascular risk. Hence, based on a large, prospective cohort study, we evaluated the impact of OSA on the risk of cardiovascular events in ACS patients with or without prior stroke.

## Methods

### Study design and population

**Fig. 1:**
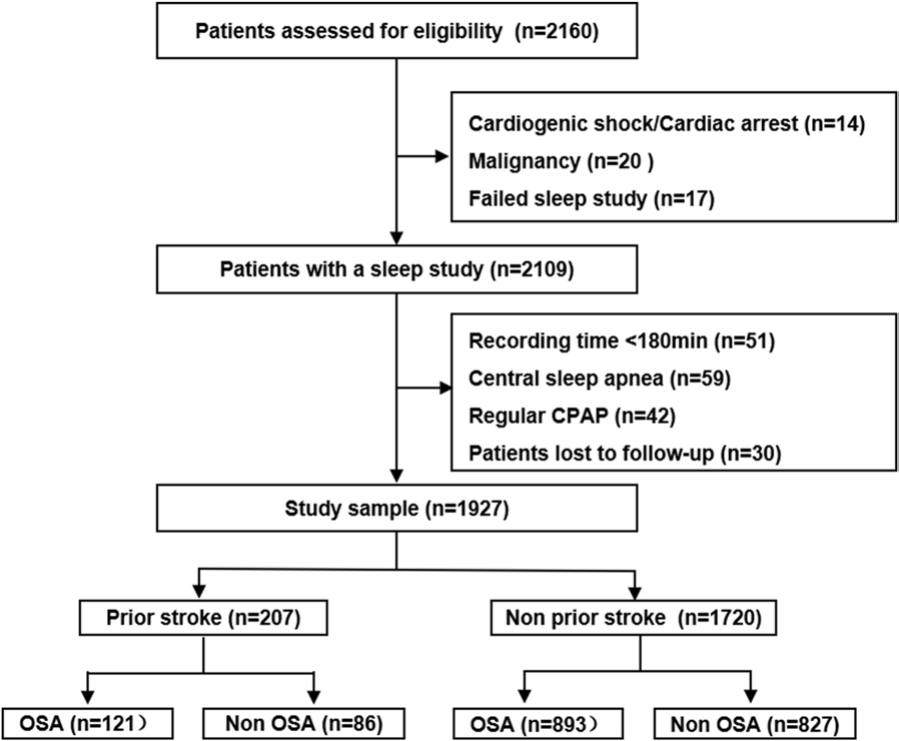
Flowchart of the study. ACS, acute coronary syndrome; CPAP, continuous positive airway pressure; OSA, obstructive sleep apnea

The OSA-ACS project (NCT03362385) is a prospective, observational, single-center study designed to evaluate the association between OSA and cardiovascular outcomes among patients with ACS. The study design has been described previously [3, 20]. In the current study, we aimed to investigate the clinical importance of OSA for patients with ACS stratified by stroke history. In brief, from June 2015 to January 2020, in the Center for Coronary Artery Disease at Beijing Anzhen Hospital, Capital Medical University, ACS patients aged 18 years to 85 years were enrolled and underwent overnight sleep studies. The exclusion criteria included cardiac arrest or cardiogenic shock, malignancies, and failed sleep studies (those who failed to obtain adequate and satisfactory recordings). Next, patients with predominantly central sleep apnea (≥ 50% central events and a central apnea–hypopnea index (AHI) ≥ 10/h), recording time < 180 min, and those receiving regular CPAP therapy (> 4 h/day and > 21 days/month) after discharge were excluded. The protocol was approved by the Ethics Committee of Beijing Anzhen Hospital, Capital Medical University (2,013,025). All participants provided written informed consent, and the study was conducted according to the amended Declaration of Helsinki. Thirty patients were lost to follow-up and therefore excluded from the analysis. The study flowchart is presented in Fig. 1.

### Overnight sleep study

For eligible patients with a hospital stay of 24–72 h, an overnight sleep study was conducted using portable cardiorespiratory polygraphy (ApneaLink Air, ResMed, Australia), which has previously been validated [21, 22]. All sleep studies were double scored manually by independent sleep somnologists blinded to the clinical characteristics, and confirmed by a senior somnologist in cases of discrepancy. They reviewed all the data and checked whether there are missed or misjudged events. The following signals were recorded: nasal airflow, thoraco-abdominal movements, snoring episodes, pulse, and percutaneous oxygen saturation (SpO_2_). Apnea was defined as an absence of airflow for 10 s or more. Hypopnea was defined as an airflow reduction of 30% for ≥ 10 s with a decrease in SpO_2_ > 4%. Oxygen desaturation was defined as a decrease in arterial oxygen saturation greater than 4%. The AHI was defined as the number of episodes of apnea hypopnea per hour of recording. The oxygen desaturation index (ODI) was calculated as the amount of time when the oxygen saturation dropped by ≥ 4% from baseline per hour of sleep. Valid tests required a minimum of 3 h of satisfactory signal recording. In accordance with previous studies, recruited patients were categorized into OSA (AHI ≥ 15 events/h) and non-OSA (AHI < 15 events/h) groups [4, 23].

### Procedures and management

Clinical care for all patients was offered at the discretion of the attending clinician based on current guidelines [24, 25]. When clinically indicated, PCI with stenting or coronary artery bypass graft (CABG) was performed. Patients with OSA, particularly those with excessive daytime sleepiness, were referred to sleep centers for further evaluation and treatment.

### Follow-up and outcomes

Following the sleep study, follow-ups were performed at one month, three months, and six months and then every six months afterwards. Clinical visits, medical chart reviews, and telephone calls by two investigators blinded to the patients' clinical information were used to collect clinical event data. At each visit, a composite of cardiovascular events was assessed, and events were then confirmed by source documentation and adjudicated by the clinical event committee.

Major adverse cardiovascular and cerebrovascular events (MACCEs), defined as a composite of cardiovascular death, MI, stroke, ischemia-driven revascularization, or hospitalization for unstable angina or heart failure, were the primary outcomes of this study. Secondary cardiovascular endpoints included the individual components of the primary composite endpoint and other composites of cardiovascular events, including cardiovascular death, MI, or stroke. The endpoints were defined according to the Standards for Data Collection in Cardiovascular Trials Initiative [26].

### Statistical analysis

Data were compared using Student's t test or the Mann–Whitney U test for continuous variables expressed as means ± standard deviations or medians (interquartile ranges). Counts and proportions (%) for categorical variables were compared using Fisher’s exact test or χ^2^ statistics as appropriate. The Kaplan–Meier product limit method was used to calculate the survival rate as the period from the initial sleep study to any MACCEs, with the data censored at the last recorded follow-up. Cox regression analysis was used to investigate independent risk factors for endpoints, and adjusted hazard ratios (HR) with 95% CI were calculated. Cox regression models were built using baseline variables that were considered clinically relevant or demonstrated a univariate relationship with the outcomes. The AHI was divided by AHI < 15, 15–30, > 30 to assess the relationship between OSA severity and the risk of cardiovascular events in patients with or without prior stroke. Multiplicative interaction terms were included in the fully adjusted models to evaluate if prior stroke modified the associations between OSA and risk of cardiovascular events. All analyses were conducted with SPSS V.26.0 (IBM SPSS, Armonk, New York, USA). A two-sided P < 0.05 was considered statistically significant.

## Results

### Baseline characteristics

Between June 2015 and January 2020, a total of 2160 patients with ACS were recruited, of whom 2109 underwent successful cardio-respiratory polygraphy. Among them, 1,969 patients with OSA and follow-up were included. Among them, only 42 (2.1%) of patients received regular CPAP therapy (> 4 h/day and > 21 days/month) and the rate was similar between those with and without prior stroke (2.8% vs. 2.1%, *P* = 0.47). A total of 1927 ACS patients met the initial eligibility criteria and 207 patients (10.74%) had a stroke history. A previous stroke was associated with older age, a less proportion of males, higher systolic blood pressure, and a higher prevalence of established risk factors, such as diabetes, hypertension, and hyperlipidemia, a lower level of Min SpO_2_, Mean SpO_2_, and a higher level of T90 SpO_2_ < 90%. Previous MI, PCI, and CABG rates were similar between the two groups (Additional file 1: Table S1).

The baseline characteristics of the OSA and non-OSA groups with or without prior stroke are shown in Table 1. Patients with OSA exhibited a higher body mass index (BMI) and neck and waist circumferences, irrespective of prior stroke. In the non-prior stroke group, patients with OSA were more males, more likely to have hypertension and prior PCI, had higher level of glycosylated hemoglobin and C-reactive protein, lower level of high-density lipoprotein, and lower left ventricular ejection fraction, and more likely to receive PCI and CABG procedures. In both the prior stroke group and the non-prior stroke group, other characteristics were generally well matched between OSA and non-OSA patients.

**Table 1:** Characteristics of the patients at baseline

Variables	All (N = 1927)	Prior stroke (n = 207)			Non-prior stroke (n = 1720)		
		OSA (n = 121)	Non-OSA(n = 86)	*P*-value	OSA (n = 893)	Non-OSA(n = 827)	*P* value
Demographics							
Age, y	56.4 ± 10.5	62.0 ± 8.9	61.6 ± 9.6	0.80	55.8 ± 10.6	55.7 ± 10.3	0.78
Male	1629 (84.5%)	100 (82.6%)	65 (75.6%)	0.21	786 (88.0%)	678 (82.0%)	< 0.001
BMI, kg/m^2^	27.1 ± 3.6	27.9 ± 3.7	25.8 ± 3.2	< 0.001	28.1 ± 3.5	26.0 ± 3.4	< 0.001
Neck, circumference, cm	41 (38–43)	41 (39 to 44)	39 (37 to 41)	< 0.001	41.5 (39 to 44)	40 (38 to 42)	< 0.001
Waist, circumference, cm	99 (93 to 105)	101 (97 to 109)	96 (90 to 100.3)	< 0.001	101 (95 to 108)	97 (91 to 102)	< 0.001
Systolic BP, mm Hg	126 (117–138)	127 (118 to 145)	130 (120 to 142)	0.47	126 (117 to 138)	126 (116 to 137)	0.26
Diastolic BP, mm Hg	76 (70–84)	76 (70 to 83.5)	75 (68.8 to 81.0)	0.64	78 (70 to 86)	75 (69 to 83)	< 0.001
Medical history							
Diabetes mellitus	609 (31.6%)	50 (58.1%)	36 (41.9%)	0.46	262 (29.3%)	254 (30.7%)	0.53
Hypertension	1247 (64.7)	103 (85.1%)	68 (79.1%)	0.26	588 (65.8%)	488 (59.0%)	0.003
Hyperlipidemia	637 (33.1)	52 (43.0%)	31 (36.0%)	0.32	291 (32.6%)	263 (31.8%)	0.73
Prior MI	316 (16.4)	23 (19.0%)	14 (16.3%)	0.61	154 (17.2%)	125 (15.1%)	0.23
Previous PCI	399 (20.7)	33 (27.3%)	16 (18.6%)	0.15	201 (22.5%)	149 (18.0%)	0.021
Previous CABG	29 (1.5)	1 (0.8%)	1 (1.2%)	0.81	17 (1.9%)	10 (1.2%)	0.25
Smoking				0.73			0.08
No	953 (49.5%)	76 (62.8%)	52 (60.5%)		442 (49.5%)	383 (46.3%)	
Yes	974 (50.5%)	45 (37.2%)	34 (39.5%)		451 (50.5%)	444 (53.7%)	
Baseline tests							
Glucose, mmol/L	5.98 (5.32 to 7.51)	6.10 (5.47 to 8.24)	6.10 (5.28 to 7.44)	0.24	6.04 (5.33 to 7.61)	5.90 (5.29 to 7.29)	0.17
Hemoglobin A1C, %	6.10 (5.60 to 7.00)	6.30 (5.60 to 7.38)	6.10 (5.70 to 7.00)	0.35	6.10 (5.70 to 7.00)	6.00 (5.60 to 6.90)	0.028
Triglyceride, mmol/L	1.51 (1.10 to 2.20)	1.44 (1.07 to 1.90)	1.38 (1.11 to 1.89)	0.53	1.59 (1.13 to 2.31)	1.47 (1.05 to 2.18)	0.16
Total Cholesterol, mmol/L	4.12 (3.47 to 4.91)	3.93 (3.40 to 4.50)	3.92 (3.46 to 4.80)	0.47	4.18 (3.52 to 4.94)	4.09 (3.41 to 4.96)	0.35
HDL-C, mmol/L	1.00 (0.86 to 1.16)	0.98 (0.87 to 1.14)	1.01 (0.84 to 1.17)	0.80	0.98 (0.85 to 1.13)	1.02 (0.87 to 1.18)	0.003
LDL-C, mmol/L	2.43 (1.90 to 3.09)	2.34 (1.92 to 2.88)	2.31 (1.90 to 3.00)	0.72	2.46 (1.95 to 3.11)	2.42 (1.83 to 3.11)	0.17
Hs-CRP, mmol/L	2.06 (0.82 to 6.13)	2.35 (0.86 to 9.25)	1.50 (0.65 to 3.98)	0.24	2.53 (1.05 to 7.24)	1.44 (0.61 to 4.69)	< 0.001
LVEF, %	61.0 (56.0 to 65.0)	62.0 (55.5 to 65.5)	58.0 (66.0 to 66.0)	0.86	60.0 (55.0 to 65.0)	62.0 (57.0 to 66.0)	0.010
Diagnosis				0.96			0.013
Unstable angina	1132 (58.7%)	74 (61.2%)	54 (62.8%)		498 (55.8%)	506 (61.2%)	
NSTEMI	365 (18.9%)	23 (19.0%)	15 (17.4%)		168 (18.8%)	159 (19.2%)	
STEMI	430 (22.3%)	24 (19.8%)	17 (19.8%)		227 (25.4%)	162 (19.6%)	
Procedures							
Vessels				0.64			0.19
0	170 (8.8%)	9 (7.4%)	7 (7.8%)		69 (7.7%)	85 (10.3%)	
1	512 (26.6%)	21 (17.4%)	19 (22.1%)		240 (26.9%)	232 (28.1%)	
≥ 2	1245 (64.6%)	91 (75.2%)	60 (69.8%)		584 (65.4%)	510 (61.7%)	
PCI	1209 (62.7%)	72 (59.5%)	52 (60.5%)	0.89	595 (66.6%)	490 (59.3%)	0.002
CABG	130 (6.7%)	14 (11.6%)	10 (11.6%)	0.99	45 (5.0%)	61 (7.4%)	0.044
Medications on discharge							
Aspirin	1877 (97.4%)	114 (94.2%)	81 (94.2%)	0.99	873 (97.8%)	809 (97.8%)	0.93
P_2_Y_12_ inhibitors	1768 (91.7%)	110 (90.9%)	80 (93.0%)	0.59	828 (92.7%)	750 (90.7%)	0.13
β-blockers	1488 (77.2%)	92 (76.0%)	52 (60.5%)	0.016	707 (79.2%)	637 (77.0%)	0.28
ACEIs/ARBs	1195 (62.0%)	82 (67.8%)	55 (64.0%)	0.57	583 (65.3%)	475 (57.4%)	0.001
Statins	1897 (98.4%)	119 (98.3%)	83 (96.5%)	0.40	878 (98.3%)	817 (98.8%)	0.42

The data is presented as mean ± SD, median (first quartile to third quartile), or n (%)

*ACEIs* Angiotensin-Converting Enzyme Inhibitors, *ARBs* angiotensin-receptor blockers, *BMI* body mass index, *BP* blood pressure, *CAD* coronary artery disease, *CABG* coronary artery bypass grafting, *LVEF* left ventricular ejection fraction, *MI* myocardial infarction, *OSA* obstructive sleep apnea, *PCI* percutaneous coronary intervention

### Results of the sleep study

The characteristics differed significantly between the two groups. The prevalence of OSA was 52.6%, and patients with OSA had a lower minimum oxygen saturation than those without OSA. Further information is shown in Table 2.

**Table 2:** Results of sleep study

Variables	All (N = 1297)	Prior stroke (n = 207)	Non-prior stroke (n = 1720)	*P* value	OSA (n = 893)	Non-OSA(n = 827)	*P* value
		OSA (n = 121)	Non-OSA(n = 86)				
AHI, events/h	16.0 (8.0 to 30.0)	29.6 (23.1 to 40.7)	7.6 (4.2 to 10.2)	< 0.001	28.8 (20.6 to 42.30	7.7 (4.1 to 10.8)	< 0.001
ODI, events/h	16.2 (8.8 to 28.6)	27.3 (21.3 to 36.4)	8.9 (5.9 to 11.5)	< 0.001	27.6 (20.1 to 40.0)	8.5 (4.8 to 11.9)	< 0.001
Min SpO_2_, %	85 (81 to 88)	80 (76 to 85)	87 (85 to 89)	< 0.001	83 (78 to 86)	88 (84 to 90)	< 0.001
Mean SpO_2_, %	94 (93 to 95)	93 (92 to 94)	94 (93 to 95)	< 0.001	93 (92 to 94)	94 (93 to 95)	< 0.001
T90 SpO_2_ < 90%	2.3 (0.4 to 10.0)	7.7 (3.0 to 23.0)	1.0 (0.2 to 5.2)	< 0.001	6.0 (2.0 to 15.0)	0.5 (0.0 to 3.0)	< 0.001
Epworth Sleepiness Scale	7.0 (4.0 to 11.0)	8.6 (6.0 to 12.0)	6.8 (3.0 to 11)	0.036	8.3 (5.0 to 12.0)	7.0 (3.0–10.0)	< 0.001

The data is presented as median (first quartile to third quartile)

*AHI* indicates apnea–hypopnea index, *ODI* oxygen desaturation index, *OSA* obstructive sleep apnea, *SpO*^*2*^ percutaneous oxygen saturation, *T90 SpO*^*2*^ percentage of total sleep time with saturation < 90%

### Outcomes in the overall population according to OSA and prior stroke

The mean median follow-up time was 2.9 years (1.5 to 3.6). Among patients with ACS, the presence of OSA was associated with a higher rate of MACCE compared with patients without OSA in the overall population (log-rank, *P* = 0.004). Kaplan–Meier analysis showed that the cumulative incidence of MACCEs was significantly higher in the prior stroke group than in the non-prior stroke group (log-rank, *P* < 0.001; Additional file 1: Fig. S1). After adjustment for the baseline risk of cardiovascular events, patients with prior stroke were strongly associated with a higher rate of MACCEs than patients without prior stroke (HR: 1.49, 95% CI: 1.12–1.98, *P* = 0.007). Furthermore, the patients were classified into three different groups based on their AHI: no/mild OSA (AHI < 15), moderate OSA (15 ≤ AHI ≤ 30), and severe OSA (30 < AHI). The association between OSA and MACCE among patients with prior stroke remained significant in moderate and severe OSA compared with the no/mild OSA (Additional file 1: Fig. S2).

### Outcomes of OSA versus non-OSA patients stratified by prior stroke

**Fig. 2:**
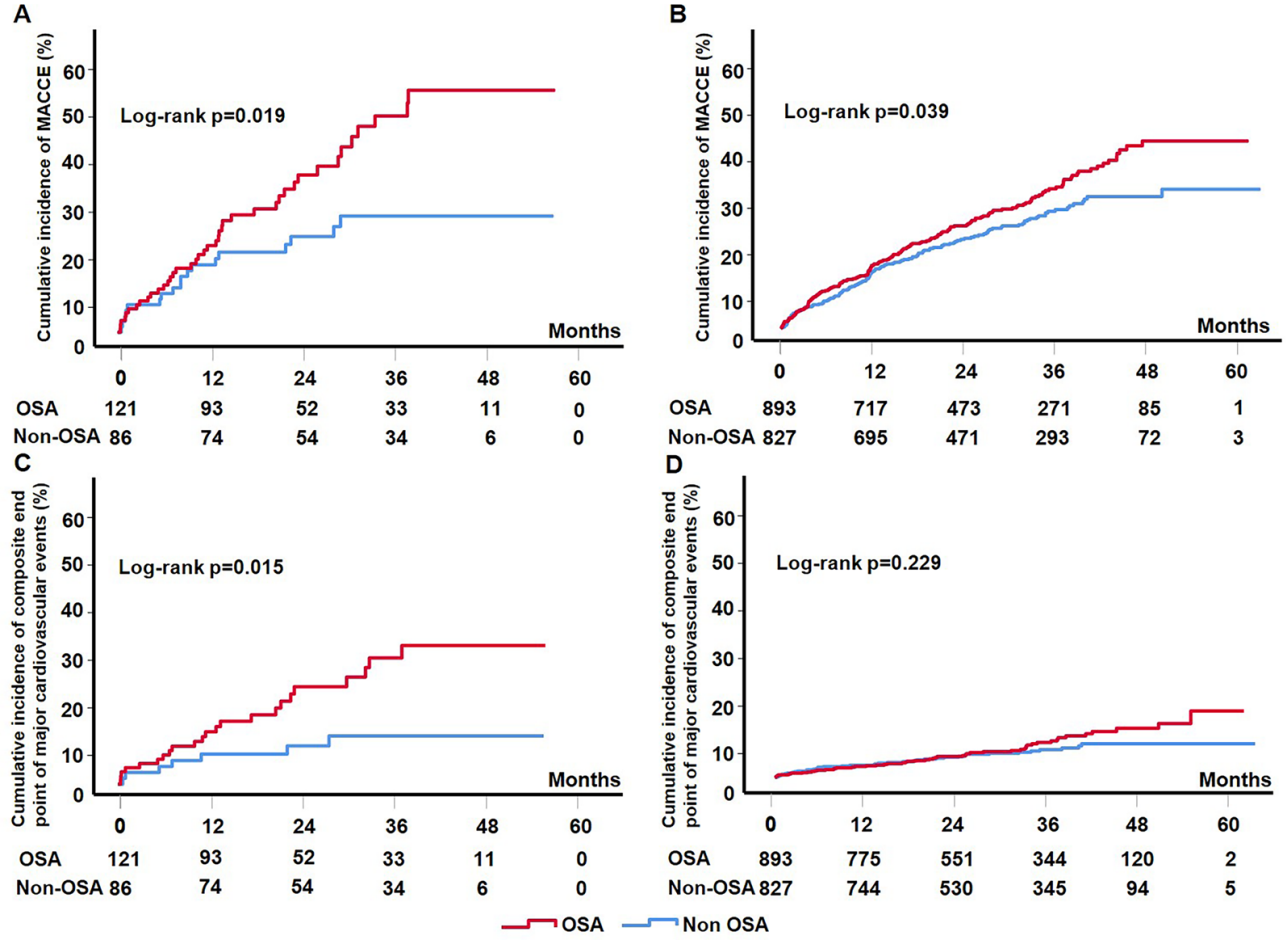
Kaplan–Meier curves for the analysis cardiovascular events in OSA versus non-OSA groups in prior stroke group and non-prior stroke group. Kaplan–Meier estimates hospitalization for MACCE (**A**, **B**) and composite of cardiovascular death, myocardial infarction, and stroke (**C**, **D**) between OSA and non-OSA groups in prior stroke group (**A**, **C**) and non-prior stroke group (**B**, **D**). MACCE, major adverse cardiovascular and cerebrovascular event. OSA, obstructive sleep apnea

In the prior stroke group, Kaplan–Meier analysis showed that MACCEs were significantly more common in the OSA group than in the non-OSA group (log-rank, *P* = 0.019; Fig. 2A). In the non-prior stroke group, the incidence of MACCEs was also higher in the OSA group than in the non-OSA group (log-rank, *P* = 0.039; Fig. 2B). After adjustment for age, sex, body mass index, smoking status, hypertension, diabetes mellitus and dyslipidemia, OSA was associated with an increased risk of MACCEs in the prior stroke group (HR: 2.04, 95% CI: 1.13–3.69, *P* = 0.018) but not in the non-prior stroke group (HR: 1.21, 95% CI: 0.96–1.52, *P* = 0.10) (Table 3). No significant interaction was noted between prior stroke and OSA for MACCE (interaction P = 0.17).

**Table 3:** Cox regression analyses evaluating the association between OSA and risk of cardiovascular events by a history of stroke

Variables	Prior stroke (n = 207)				Non-prior stroke (n = 1720)			
	Unadjusted HR (95% CI)	*P*-value	Adjusted HR^a^ (95% CI)	*P*-value	Unadjusted HR (95% CI)	*P* value	Adjusted HR^a^ (95% CI)	*P* value
MACCE	1.93 (1.10 to 3.36)	0.021	2.04 (1.13 to 3.69)	0.018	1.26 (1.01 to 1.56)	0.040	1.21 (0.96 to 1.52)	0.10
Cardiovascular death	1.15 (0.32 to 4.06)	0.83	1.09 (0.28 to 4.23)	0.90	1.23 (0.54 to 2.80)	0.63	1.05 (0.44 to 2.49)	0.91
Myocardial infarction	4.15 (0.48 to 35.6)	0.19	6.61 (0.61 to 71.56)	0.12	1.55 (0.85 to 2.84)	0.15	1.44 (0.76 to 2.74)	0.27
Stroke	3.22 (0.91 to 11.4)	0.07	3.03 (0.82 to 11.25)	0.10	0.83 (0.40 to 1.75)	0.62	0.84 (0.39 to 1.83)	0.67
Ischemia-driven revascularization	1.57 (0.63 to 3.89)	0.33	1.62 (0.62 to 4.23)	0.33	1.32 (0.94 to 1.85)	0.11	1.27 (0.89 to 1.82)	0.19
Hospitalization for unstable angina	1.37 (0.65 to 2.87)	0.41	1.42 (0.65 to 3.11)	0.38	1.25 (0.97 to 1.61)	0.09	1.21 (0.92 to 1.58)	0.17
Hospitalization for heart failure	1.16 (0.19 to 6.98)	0.87	0.96 (0.14 to 6.80)	0.97	0.95 (0.36 to 2.53)	0.92	0.81 (0.29 to 2.26)	0.69
Composite of major cardiovascular events^b^	2.73 (1.17 to 6.38)	0.020	2.84 (1.17 to 6.93)	0.022	1.29 (0.86 to 1.95)	0.23	1.19 (0.77 to 1.84)	0.44
All-cause death	0.67 (0.22 to 1.99)	0.47	0.67 (0.21 to 2.17)	0.50	1.01 (0.51 to 1.99)	0.99	0.86 (0.42 to 1.75)	0.68
All repeat revascularization	1.65 (0.74 to 3.65)	0.22	1.87 (0.81 to 4.34)	0.14	1.18 (0.89 to 1.55)	0.26	1.11 (0.83 to 1.49)	0.49

*MACCE*, major adverse cardiovascular and cerebrovascular event including cardiovascular death, MI, stroke, ischemia-driven revascularization, or hospitalization for unstable angina or heart failure. *OSA* obstructive sleep apnea

^a^Model adjusted for age, sex, BMI, smoker, hypertension, diabetes mellitus and dyslipidemia

^b^Composite end point of major cardiovascular events included cardiovascular death, myocardial infarction, and stroke

Among patients with prior stroke, Kaplan–Meier analysis showed that the cumulative incidence of composite events of cardiovascular death, MI, or stroke was significantly higher in the OSA group than in the non-OSA group (log-rank, *P* = 0.015; Fig. 2C) but not among patients with non-prior stroke (log-rank, *P* = 0.23; Fig. 2D). The fully adjusted multivariable Cox regression model analysis showed that the association between OSA and the outcomes remained statistically significant in the prior stroke group (HR: 2.84, 95% CI: 1.17–6.93, *P* = 0.022) but not in the non-prior stroke group (HR: 1.19, 95% CI: 0.78–1.84, *P* = 0.44). The other endpoints were not different and are listed in Table 3. The crude numbers of events are listed in Additional file 1: Table S2. We also performed additional subgroup analyses according to prior coronary artery disease, prior myocardial infarction, and prior cardiovascular diseases (myocardial infarction, history of revascularization, heart failure, or atrial fibrillation/flutter). Although differences were found between some subgroups, the association of OSA with MACCE was not modified by these confounding factors (interaction P > 0.14 for all) (Additional file 1: Table S3). Then, we calculated the MACCE rate between CPAP and non-CPAP groups in prior stroke (33.3% vs 33.9%, *P* = 0.99) and non-prior stroke groups (19.4% vs 20.8%, *P* = 0.99) and found no significant differences in both groups.

## Discussion

To our knowledge, this is the first prospective study evaluating the prognostic value of OSA in ACS patients with or without prior stroke. After adjustment for potential confounders, compared with patients without OSA, patients with OSA had a 2.0-fold higher risk of MACCE in the prior stroke group, but not in the non-prior stroke group, although no significant interaction was noted between prior stroke and OSA for MACCE (interaction P = 0.17). The incidence of composite events of cardiovascular death, MI, or stroke was also significantly higher in the OSA versus non-OSA group among patients with prior stroke but not among those without prior stroke.

Given that ACS and stroke have similar pathogeneses, such as atherosclerosis and thrombosis, it is not surprising that prior stroke plays an important role in determining ACS outcome [14, 27]. Studies have indicated that the percentage of patients with ACS and a prior stroke is approximately 10% [28]. Our findings are consistent with recent data showing that 10.74% of patients had a history of prior stroke, and previous stroke patients tended to be older, were more likely to be female, and were more likely to have diabetes, hypertension, and hyperlipidemia. Recently, a large study revealed that patients with prior stroke who undergo PCI have an increased risk of long-term cardiovascular and cerebrovascular complications, specifically recurrent strokes [13]. Similarly, we also found that prior stroke significantly predicted subsequent cardiovascular events in ACS patients. Atherosclerosis, comorbidities, cardiovascular risk factors, and low use of medical and invasive therapy might contribute to poor outcomes in patients with a history of stroke [15, 29]. Therefore, given the high incidence of prior stroke among ACS patients and its possible effect on prognosis, greater attention should be given to ACS patients with prior stroke.

Current evidence indicates that OSA is closely related to poor outcomes in patients with ACS or cerebrovascular disease [1, 4, 5]. Our previous studies also showed that OSA was closely related to poor cardiovascular outcomes in ACS onset, especially regarding diabetes status [3, 20]. Emerging evidence has also demonstrated a close relationship between OSA and stroke. OSA is more prevalent following incident ischemic strokes since insults to the central nervous system result in changes in breathing patterns or could mask previously undiagnosed pre-stroke OSA in the poststroke period [30]. Moreover, several studies have demonstrated that OSA in poststroke patients is associated with an increased risk of recurrent ischemic stroke and worse outcomes [17, 18, 31]. Patients with untreated severe OSA are twice as likely to suffer an incident stroke, and this risk is especially relevant to younger and middle-aged patients, without a difference between men and women [32, 33]. In terms of mechanism, OSA may initiate and worsen atherosclerosis via the activation of oxidative stress, inflammation, the sympathetic nervous system, and metabolic abnormalities, eventually leading to high morbidity and mortality in cerebrovascular disease [34–36]. The ESADA Cohort demonstrated the role of OSA-related hypoxia in the risk of developing cardioembolic complications such as stroke [37]. Thus, OSA and stroke patients with ACS must be given extra attention, as they may exhibit a synergistic deleterious effect that increases future cardiovascular risk.

The benefits of CPAP therapy are well recognized: it eliminates obstructive events during sleep and substantially improves the consequences, especially daytime sleepiness, neurocognitive deficits and driving performance. However, in randomized controlled trials, the use of CPAP was not associated with a reduction in cardiovascular outcomes among patients with ACS [7–10]. Given that the phenotype of patients is not homogeneous, the deleterious effects of OSA could be different depending on the specific subgroups of ACS patients [38]. Therefore, it is important to focus on identifying specific subgroups of patients with ACS and reevaluate the effect of OSA treatment on cardiovascular diseases [39]. In particular, there is evidence suggesting that CPAP may improve sleepiness, neurological recovery, and depressive symptoms post-stroke in stroke survivors with OSA [19]. In our study, patients with OSA and prior stroke were at the increased risk of incurring a MACCE, therefore representing a high-risk subset most likely to respond to the intervention. There is no significant interaction between prior stroke and OSA for the combined or individual cardiovascular events, possibly due to the lack of power as a result of the small proportion of prior stroke group in this cohort and small number of events in a relatively short follow-up duration. This finding invites us to consider the possibility that a deleterious OSA effect, which was not observed in the entire population that suffered an ACS, exists in this specific phenotype. In contrast, the ancillary study of the ISAACC study showed that OSA was associated with an increased risk of recurrent cardiovascular events in patients without previous heart disease [23]. The variability of results might be partly explained by racial differences and suggests potential heterogeneity of ACS phenotype. We recruited predominantly East Asian patients, which cannot be generalized to patients with other ethnic or racial backgrounds. Noteworthy, patients in our study had more traditional risk factors and more severe daytime sleepiness than those in the ISAACC study [23]. Additionally, patients with prior stroke represents a high-risk subgroup with more female and more than 30% higher prevalence of all 3 traditional risk factors (hypertension, diabetes, hyperlipidemia), and had higher long-term events rate than those without prior stroke. Coexistence of these factors with OSA may generate synergistic effects and promote progression of lesions, thus increasing ischemic events in the long run [20, 40].

The premise of precision medicine is to use a variety of tools to differentiate an individual patient from other patients with similar clinical presentations and thus tailor treatments to that patient's particular needs. Hence, patients with ACS who previously had a stroke should be screened for OSA, and interventions may be necessary. Moreover, more OSA trials should be performed in this subgroup to determine the effects of OSA treatment.

## Limitations

This study has several potential limitations. First, the prior stroke cohort included 207 patients, which may diminish the generalizability of the prognostic value. Second, the diagnosis of OSA based on portable sleep monitors may underestimate the severity of OSA. However, studies have shown that portable polygraphy can be used as an alternative to polysomnography for OSA diagnosis [41]. Third, patients self-reported their prior stroke history, which could result in some bias, and we could not confirm whether the strokes were hemorrhagic or ischemic. Therefore, the patients in our study received professional assistance to obtain admission information and conduct grouping, minimizing this bias. Finally, this study recruited predominantly East Asian patients, so it cannot be generalized to patients with other ethnic or racial backgrounds.

## Conclusions

Among ACS patients, the presence of OSA was associated with an increased risk of cardiovascular events in patients with prior stroke. Further trials exploring the efficacy of OSA treatment in high-risk patients with ACS and prior stroke are warranted.

## Supplementary Information

**Additional file 1: Table S1.** Baseline patient characteristics. **Table S2.** Crude Number of all Events by Prior Stroke Categories. **Table S3.** Association of OSA with risk of MACCE according to subgroups. **Fig. S1.** Kaplan-Meier curves for the analysis of cardiovascular events in prior stroke versus non-prior stroke group. **Fig. S2.** Kaplan-Meier curves for the analysis of cardiovascular events according to obstructive sleep apnea severity in prior stroke (A) versus non-prior stroke group (B).

## Data Availability

The datasets used and/or analysed during the current study available from the corresponding author on reasonable request.

## Abbreviations

ACS: Acute coronary syndrome
AHI: Apnea–hypopnea index
BMI: Body mass index
CPAP: Continuous positive airway pressure
MACCE: Major adverse cardiovascular and cerebrovascular event
NSTEMI: Non-ST-segment elevation myocardial infarction
OSA: Obstructive sleep apnea
STEMI: ST-segment elevation myocardial infarction
