# Supplementary material for: Clinical significance of obstructive sleep apnea in patients with acute coronary syndrome with or without prior stroke: a prospective cohort study
Source: Eur J Med Res. 2023 Mar 1;28:107. doi: 10.1186/s40001-023-01071-0 (PMC9976418; doi:10.1186/s40001-023-01071-0)

**Table S1. Baseline patient characteristics.**

| **Variables** | **Prior stroke group n=207** | **Non-prior stroke group n=1720** | ***P* value** |
| --- | --- | --- | --- |
| **Demographics** |  |  |  |
| Age, y | 61.8 ± 9.2 | 55.7 ± 10.4 | ＜0.001 |
| Male | 165 (79.7%) | 1464 (85.1%) | 0.04 |
| BMI, kg/m^2^ | 27.1 ± 3.6 | 27.1 ± 3.6 | 0.17 |
| Neck, circumference, cm | 40 (38 to 43) | 41 (38 to 43) | 0.23 |
| Waist, cm | 99 (93.5 to 106) | 99 (93 to 105) | 0.71 |
| Systolic BP, mm Hg | 130 (120 to 143) | 126 (117 to 138) | 0.028 |
| Diastolic BP, mm Hg | 76 (70 to 83) | 76 (70 to 85) | 0.99 |
| **Medical history** |  |  |  |
| Diabetes mellitus | 93 (44.9%) | 516 (30.0%) | ＜0.001 |
| Hypertension | 171 (82.6%) | 1076 (62.6%) | ＜0.001 |
| Hyperlipidemia | 83 (40.1%) | 554 (32.2%) | 0.023 |
| Prior myocardial infarction | 37 (17.9%) | 279 (16.2%) | 0.54 |
| Previous PCI | 49 (23.7%) | 350 (20.3%) | 0.27 |
| Previous CABG | 2 (1.0%) | 27 (1.6%) | 0.50 |
| Smoking |  |  | 0.004 |
| No | 128 (61.8%) | 839 (48.8%) |  |
| Yes | 79 (38.2%) | 881 (51.2%) |  |
| **Baseline tests** |  |  |  |
| Glucose, mmol/L | 6.15 (5.41 to 7.66) | 5.97 (5.31 to 7.44) | 0.27 |
| Hemoglobin A1C, % | 6.1 (5.7 to 7.3) | 6.0 (5.6 to 7.0) | 0.11 |
| Triglyceride, mmol/L | 1.43 (1.10 to 1.89) | 1.53 (1.11 to 2.24) | 0.83 |
| Total Cholesterol, mmol/L | 3.92 (3.42 to 4.59) | 4.15 (3.48 to 4.95) | 0.96 |
| HDL-C, mmol/L | 1.00 (0.86 to 1.15) | 1.00 (0.86 to 1.16) | 0.44 |
| LDL-C, mmol/L | 2.33 (1.92 to 2.90) | 2.45 (1.90 to 3.11) | 0.99 |
| Hs-CRP, mmol/L | 2.14 (0.77 to 6.47) | 1.98 (0.78 to 6.08) | 0.86 |
| Uric, mmol/L | 359 (291 to 426) | 362 (301 to 426) | 0.25 |
| LVEF, % | 62 (56 to 66) | 61 (56 to 65) | 0.43 |
| **Diagnosis** |  |  | 0.59 |
| Unstable angina | 128 (61.8%) | 1004 (58.4) |  |
| NSTEMI | 38 (18.4%) | 327 (19.0%) |  |
| STEMI | 41 (19.8%) | 389 (22.6%) |  |
| **Procedures** |  |  |  |
| Vessels |  |  | 0.025 |
| 0 | 16 (7.7%) | 154 (9.0%) |  |
| 1 | 40 (19.3%) | 472 (27.4%) |  |
| ≥2 | 151 (72.9%) | 1094 (63.6%) |  |
| PCI | 124 (59.9%) | 1085 (63.1%) | 0.37 |
| CABG | 24 (11.6%) | 106 (6.2%) | 0.003 |
| **Medications on discharge** |  |  |  |
| Aspirin | 195 (94.2%) | 1682 (97.8%) | 0.002 |
| P_2_Y_12_ inhibitors | 190 (91.8) | 1578 (91.7%) | 0.98 |
| β-blockers | 144 (69.6%) | 1344 (78.1%) | 0.005 |
| ACEIs/ARBs | 137 (66.2%) | 1058 (61.5%) | 0.09 |
| Statins | 202 (97.6) | 1695 (98.5%) | 0.29 |
| **Sleep study parameters** |  |  |  |
| AHI, events/h | 18.8 (8.5 to 32.9) | 15.7 (7.9 to 29.6) | 0.08 |
| ODI, events/h | 18.4 (10.1 to 29.6) | 16.1 (8.7 to 28.3) | 0.12 |
| Min SpO_2_, % | 84 (78 to 87) | 85 (81 to 88) | ＜0.001 |
| Mean SpO_2_, % | 93 (92 to 95) | 94 (92 to 95) | 0.003 |
| T90 SpO_2_<90% | 4.3 (1.0 to 13.3) | 2.0 (0.3 to 9.7) | ＜0.001 |
| Epworth Sleepiness Scale | 7.0 (4.0 to 11.0) | 7.0 (4.0 to 11.0) | 0.70 |

Data are presented as mean ± SD, median (first quartile to third quartile), or n (%).

Abbreviations: ACEIs, Angiotensin-Converting Enzyme Inhibitors; ARBs, angiotensin-receptor blockers; BMI, body mass index; BP, blood pressure; CAD, coronary artery disease; CABG, coronary artery bypass grafting; Hs-CRP, high-sensitivity C-reactive protein; LVEF, left ventricular ejection fraction; MI, myocardial infarction; NSTEMI, non-ST-segment elevation myocardial infarction; OSA, obstructive sleep apnea; PCI, percutaneous coronary intervention. SpO_2_, percutaneous oxygen saturation, STEMI, ST-segment-elevation myocardial infarction.

**Table S2. Crude Number of all Events by Prior Stroke Categories.**

| **Variables** | **Prior stroke group (n=207)** | | **Non-prior stroke group (n=1720)** | |
| --- | --- | --- | --- | --- |
|  | **OSA (n=121, 58.5%)** | **Non-OSA (n=86, 41.5%)** | **OSA (n=893, 51.9%)** | **Non-OSA (n=827, 48.1%)** |
| MACCE | 41(33.9%) | 18(20.9%) | 186(20.8%) | 144(17.4%) |
| Cardiovascular death | 6(5.0%) | 4(4.7%) | 11(1.3%) | 12(1.6%) |
| Myocardial infarction | 5(4.1%) | 1(1.2%) | 136(15.2%) | 106(12.8%) |
| Stroke | 3(2.5%) | 2(2.3%) | 13(1.5%) | 15(1.8%) |
| Ischemia-driven revascularization | 14(11.6%) | 7(8.1%) | 80 (9.0%) | 58(7.0%) |
| Hospitalization for unstable angina | 19(15.7%) | 11(12.8%) | 136(15.2%) | 106(12.8%) |
| Hospitalization for heart failure | 19(15.7%) | 11(12.8%) | 8(0.9%) | 8(1.0%) |
| Composite of major cardiovascular events | 23 (19.0%) | 7 (8.1%) | 53 (5.9%) | 39 (4.7%) |
| Composite for cardiac events | 30 (24.8%) | 16 (18.6%) | 175 (19.6%) | 132 (16.0) |
| All repeat revascularization | 23 (13.0%) | 15 (10.8%) | 110 (12.3%) | 89 (10.8%) |
| All death | 6(5.0%) | 7(8.1%) | 13(1.5%) | 10(1.2%) |

Composite end point of major cardiovascular events included cardiovascular death, myocardial infarction, and stroke; Composite for cardiac events included cardiovascular death, myocardial infarction, ischemia-driven revascularization, or hospitalization for unstable angina or heart failure. CI, confidence interval; MACCE, major adverse cardiovascular and cerebrovascular event; OSA, obstructive sleep apnea

**Table S3. Association of OSA with risk of MACCE according to subgroups.**

| **Subgroups** | **OSA (n=1,014)** | **Non-OSA (n=913)** | **Adjusted HR (95% CI) *** | **P value** | **P for interaction** |
| --- | --- | --- | --- | --- | --- |
| Prior CAD |  |  |  |  |  |
| Yes | 100/445 (22.5) | 67/372 (18.0) | 1.37 (0.99, 1.89) | 0.06 | 0.91 |
| No | 127/569 (22.3) | 95/541 (17.6) | 1.21 (0.91, 1.60) | 0.18 |  |
| Prior MI |  |  |  |  |  |
| Yes | 50/177 (28.1) | 24/139 (17.3) | 1.74 (1.04, 2.90) | 0.03 | 0.14 |
| No | 177/837 (21.1) | 138/774 (17.8) | 1.19 (0.94, 1.51) | 0.15 |  |
| Prior Cardiovascular diseases |  |  |  |  |  |
| Yes | 76/316 (24.1) | 45/239 (18.8) | 1.26 (0.98, 1.63) | 0.08 | 0.80 |
| No | 151/698 (21.6) | 117/674 (17.4) | 1.31 (0.89, 1.95) | 0.17 |  |

Data are presented as n/N (%). MI, myocardial infarction; CAD, coronary artery disease; CI, confidence interval; HR, hazard ratio; MACCE, major adverse cardiovascular and cerebrovascular event; OSA, obstructive sleep apnea; *****Adjusted for age, sex, body mass index, smoking, hypertension, diabetes mellitus, hyperlipidemia, prior stroke, and clinical presentation (unstable angina [referent], acute myocardial infarction). Prior Cardiovascular diseases included prior myocardial infarction, history of revascularization, heart failure, atrial fibrillation/flutter.

Fig. S1 Kaplan-Meier curves for the analysis of cardiovascular events in prior stroke versus non-prior stroke group

~~
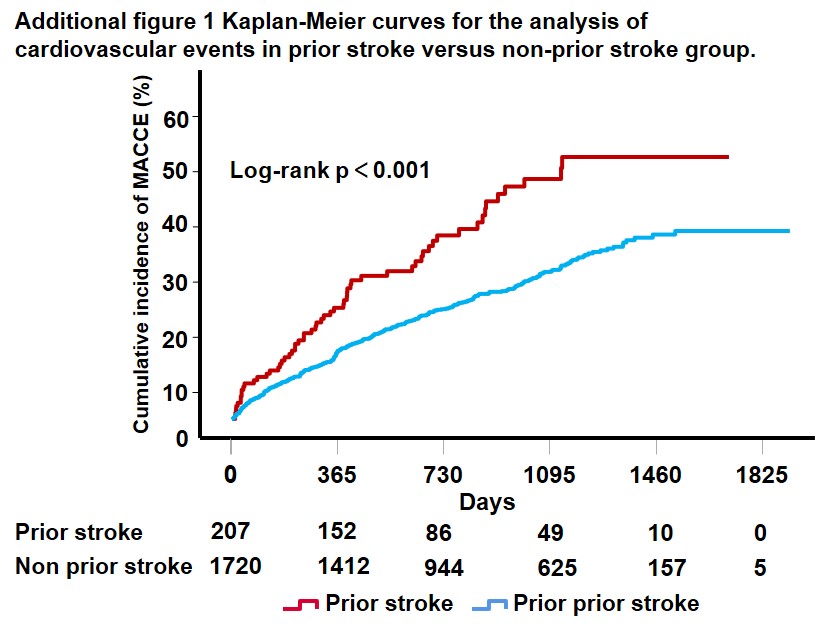
~~


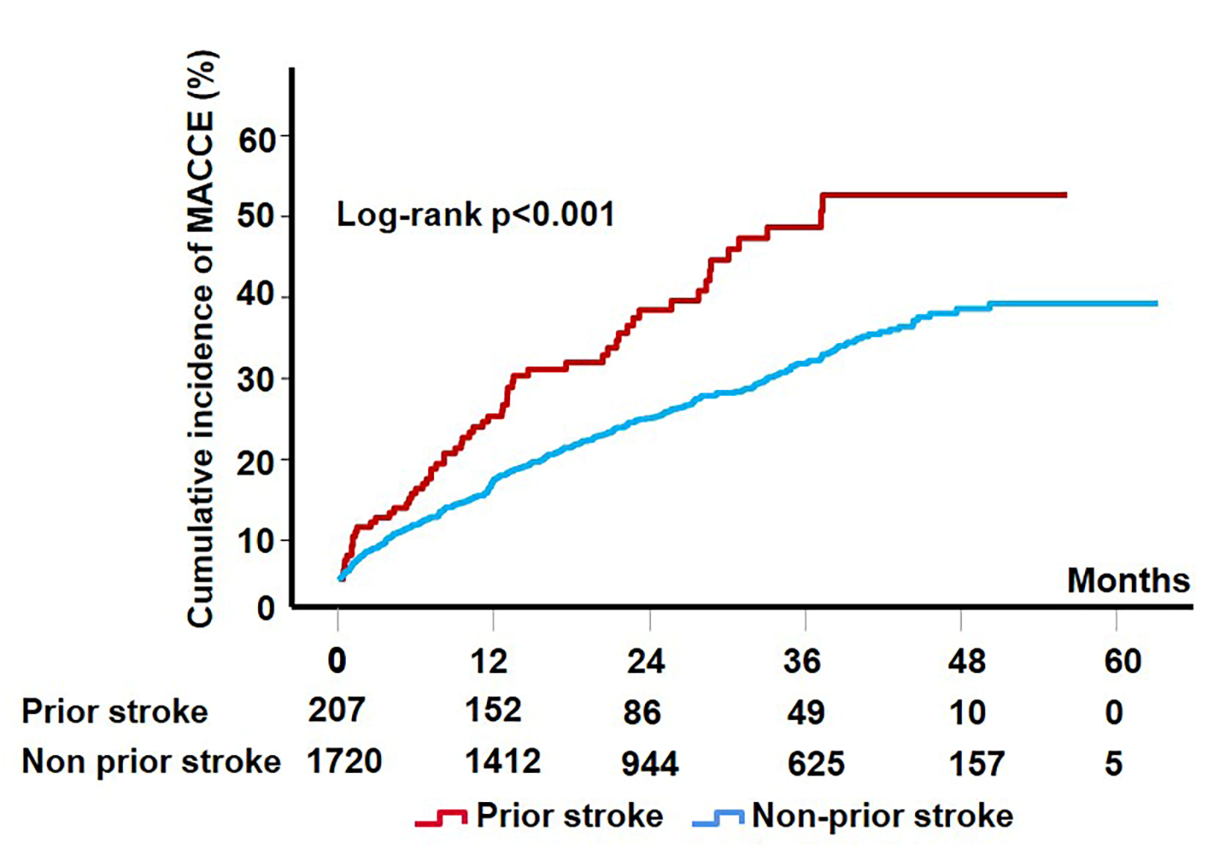


Fig. S2 Kaplan-Meier curves for the analysis of cardiovascular events according to obstructive sleep apnea severity in prior stroke (A) versus non-prior stroke group (B)


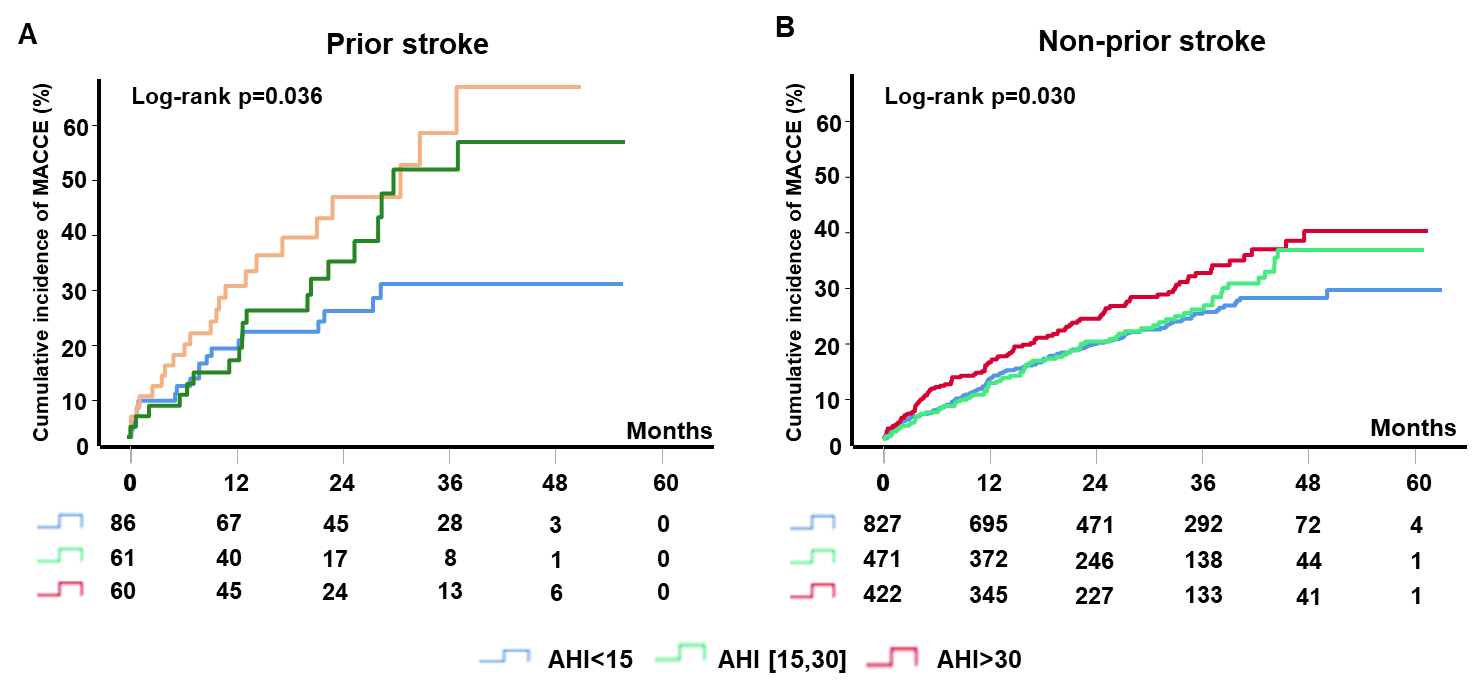

Supplement: Supplementary file 1 — Additional file 1: Table S1. Baseline patient characteristics. Table S2. Crude Number of all Events by Prior Stroke Categories. Table S3. Association of OSA with risk of MACCE according to subgroups. Fig. S1. Kaplan-Meier curves for the analysis of cardiovascular events in prior stroke versus non-prior stroke group. Fig. S2. Kaplan-Meier curves for the analysis of cardiovascular events according to obstructive sleep apnea severity in prior stroke (A) versus non-prior stroke group (B). [file 40001_2023_1071_MOESM1_ESM.docx]
